# ARE TRACK-AND-TRIGGER SYSTEMS GOOD NEWS FOR RESIDENTS IN LONG-TERM CARE FACILITIES? A MULTI-METHOD EVALUATION

**DOI:** 10.1093/geroni/igz038.1878

**Published:** 2019-11-08

**Authors:** Robert O Barker, Siân Russell, Rachel Stocker, Jennifer Liddle, Joy Adamson, Barbara Hanratty

**Affiliations:** Newcastle University, Newcastle upon Tyne, United Kingdom

## Abstract

Changes in physiological measurements such as blood pressure or temperature may signal deteriorating health before it is apparent. Early warning (or track and trigger) systems provide a framework to use such measurements to identify acute illness and prompt a timely response. They are in widespread use in acute hospitals across North America and Europe, but few have been validated in community settings. Hospitals in the UK have adopted the National Early Warning Score (NEWS) which measures temperature, respiratory rate, pulse, blood pressure, oxygen saturation and conscious level. This presentation describes a multi-method evaluation of the introduction of NEWS into 47 long-term care facilities. Staff with little or no healthcare training were tasked with digital recording of the NEWS. This multi-method evaluation consisted of a survey to explore staff views (n=42), a quantitative analysis of approximately 17,000 NEWS readings, and 21 semi-structured qualitative interviews with stakeholders. Survey and interview findings suggested that use of the score increased staff confidence in communication and care. There were many challenges to implementation, including practical difficulties in measuring vital signs, competing priorities for staff and a persistent lack of shared understanding across professional boundaries. Quantitative analysis of recorded scores described an increase in use of the NEWS over time, but wide variation in uptake between different facilities. Early warning systems may enhance management of acute illness in long-term care facilities but implementation is not straightforward. This presentation will discuss will discuss findings in depth - what worked, lessons learned and implications for the future.

